# Minimal Holocene retreat of large tidewater glaciers in Køge Bugt, southeast Greenland

**DOI:** 10.1038/s41598-017-12018-x

**Published:** 2017-09-26

**Authors:** Laurence M. Dyke, Camilla S. Andresen, Marit-Solveig Seidenkrantz, Anna L. C. Hughes, John F. Hiemstra, Tavi Murray, Anders A. Bjørk, David A. Sutherland, Flor Vermassen

**Affiliations:** 1 Geological Survey of Denmark and Greenland, Department of Glaciology and Climate, Øster Voldgade 10, DK-1350 København K, Denmark; 2 Centre for Past Climate Studies, Department of Geoscience, Aarhus University, Høegh-Guldbergs Gade 2, DK-8000 Aarhus C, Denmark; 3 Department of Earth Science, University of Bergen and Bjerknes Centre for Climate Research, Allégaten 41, N-5007 Bergen, Norway; 4 Glaciology Group, Swansea University, Singleton Park, Swansea, SA2 8PP UK; 5 Centre for GeoGenetics, Natural History Museum of Denmark, University of Copenhagen, Øster Voldgade 5–7, DK-1350 København K, Denmark; 6 Department of Geological Sciences, 1272 University of Oregon, Eugene, OR 97403-1272 USA

## Abstract

Køge Bugt, in southeast Greenland, hosts three of the largest glaciers of the Greenland Ice Sheet; these have been major contributors to ice loss in the last two decades. Despite its importance, the Holocene history of this area has not been investigated. We present a 9100 year sediment core record of glaciological and oceanographic changes from analysis of foraminiferal assemblages, the abundance of ice-rafted debris, and sortable silt grain size data. Results show that ice-rafted debris accumulated constantly throughout the core; this demonstrates that glaciers in Køge Bugt remained in tidewater settings throughout the last 9100 years. This observation constrains maximum Holocene glacier retreat here to less than 6 km from present-day positions. Retreat was minimal despite oceanic and climatic conditions during the early-Holocene that were at least as warm as the present-day. The limited Holocene retreat of glaciers in Køge Bugt was controlled by the subglacial topography of the area; the steeply sloping bed allowed glaciers here to stabilise during retreat. These findings underscore the need to account for individual glacier geometry when predicting future behaviour. We anticipate that glaciers in Køge Bugt will remain in stable configurations in the near-future, despite the predicted continuation of atmospheric and oceanic warming.

## Introduction

Southeast Greenland has experienced dramatic glaciological changes over the last two decades. Glaciers across the region simultaneously retreated, accelerated, and thinned^1–4^. It is thought that this increase in ice loss was triggered by submarine melting at tidewater glacier termini, in turn caused by the warming of the subpolar North Atlantic^5,6^. However, the precise mechanisms controlling marine-terminating glacier retreat are not well-understood, largely because of the difficulties in monitoring these extremely inhospitable environments. This knowledge gap makes it difficult to accurately predict future glacier behaviour and to assess the contribution of glaciers in southeast Greenland to global sea-level rise. Recent work has also highlighted that this region of Greenland is important in modulating global thermohaline circulation; this occurs through variation in the mixing of ocean currents on the continental shelf ^7^, and through the enhanced influx of glacial meltwater^8,9^. Despite its obvious importance, relatively little is known about the glaciological and oceanographic history of southeast Greenland. Direct observations of glacier behaviour and ocean conditions only extend back to the early 20th Century^10,11^; these provide brief snapshots of ice-ocean interactions. We present results from the first sediment core obtained from Køge Bugt, the drainage portal for one of the largest glacial systems in Greenland^12^. We analyse ice-rafted debris abundance, foraminiferal assemblages, and sortable silt grain size to constrain glacier behaviour and oceanographic conditions through the last 9100 years. We use these results to assess the stability of glaciers in Køge Bugt in response to changing ocean and climatic conditions during the Holocene. These new data provide a baseline against which to assess the significance of current glaciological changes. They are also useful for improving predictions of future behaviour.

**Figure 1:**
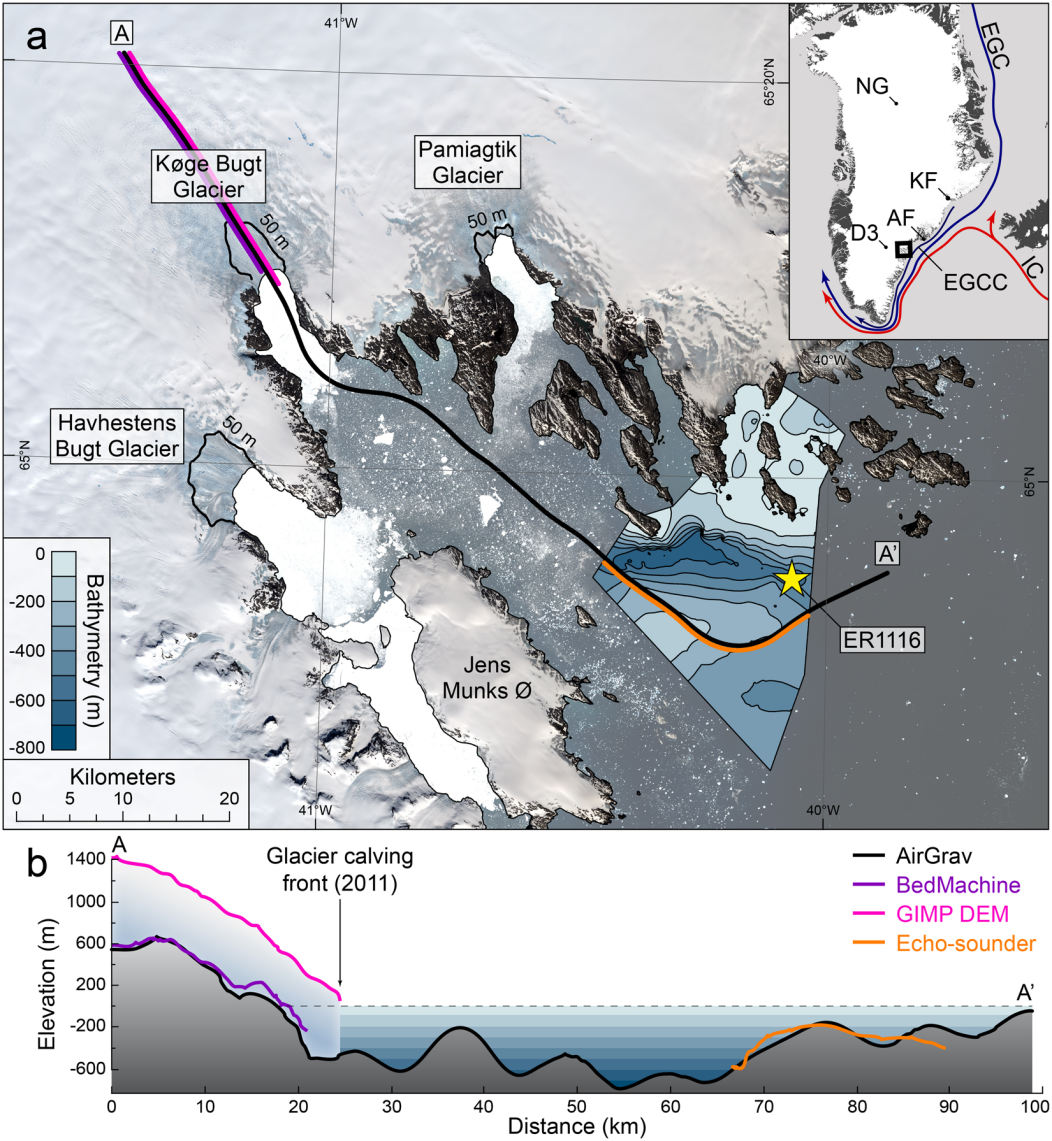
(**a**) Køge Bugt, 1:600,000. Background imagery is a mosaic of Landsat 8 scenes^65^. The star marks the location of core ER1116. The 50 m ice sheet bed contour^15^ delineates the maximum area of the ice sheet bed that was below sea-level during the early-Holocene^63^. Køge Bugt bathymetric data were interpolated from new single-beam echo-soundings presented here. The inset map shows the extent of the main figure and a schematic of ocean surface circulation^42,66^. Inset map labels: NG–NGRIP ice core, KF–Kangerdlugssuaq Fjord, AF–Ammassalik Fjord, D3–Dye 3 ice core, EGC–East Greenland Current, EGCC–East Greenland Coastal Current, and IC–Irminger Current. (**b**) Bathymetric and topographic measurements in Køge Bugt. Data from aerogravity^14^ (black), ice-penetrating radar^15,16^ (purple), satellite altimetry^67^ (magenta), and echo-sounder measurements (orange) were extracted along the transect A–A′. The spatial extent of each dataset is shown in 1a. This figure was created using *ArcMap* 10.1, *Microsoft Excel* 2013, and *Adobe Illustrator CS6*.

Køge Bugt (Ikeq) is a large glacial embayment in the centre of southeast Greenland (Fig. 1). Three major tidewater glaciers terminate in the bay, draining ice from ~27,000 km^2^ of the Greenland Ice Sheet. These glaciers are amongst the fastest in Greenland^3^; velocities near calving termini can exceed 12 km yr^−1^. Glaciers here experienced significant, dynamically-induced thinning in the early-2000s^1^. This was followed by a period of glacier stabilisation and thickening during the latter half of the 2000s^13^. Nevertheless, between 2000 and 2012 glaciers in Køge Bugt were the third largest contributor to sea-level rise from Greenland, with a collective discharge anomaly of 67 Gt^12^.

The bathymetry of Køge Bugt was previously largely unknown. New bathymetric data, although limited, highlight the presence of a deep (>700 m bsl) trough in the centre of Køge Bugt (Fig. 1). Aerogravity^14^ and subglacial topography data^15,16^ show that this glacial trough extends through the bay, but rises above sea-level only a few kilometres inland of the present-day ice margin (Fig. 1). Køge Bugt has a Polar maritime climate and receives in excess of 2000 mm w.e. of precipitation annually^17,18^; this is the highest accumulation rate in Greenland.

A small number of oceanographic measurements are available from the continental shelf offshore from Køge Bugt^19,20^ (Fig. S1). The data show a thick surface layer composed of cold Polar waters; the East Greenland Current and East Greenland Coastal Current. These currents are characterised by temperatures from 0 to 2 °C and salinities below 33 psu. Warmer, saline waters of the Irminger Current lie below this; temperatures are typically ~4 °C and salinities are ~35 psu. The only oceanographic measurements from within Køge Bugt are temperature profiles which were obtained using sensors mounted on marine mammals^21,22^. These data show a similar pattern to the continental shelf; surface waters are cold (~0 °C) to a depth of around 300 m, these are underlain by warmer (~4 °C) water masses (Fig. S1).

## Results

**Figure 2:**
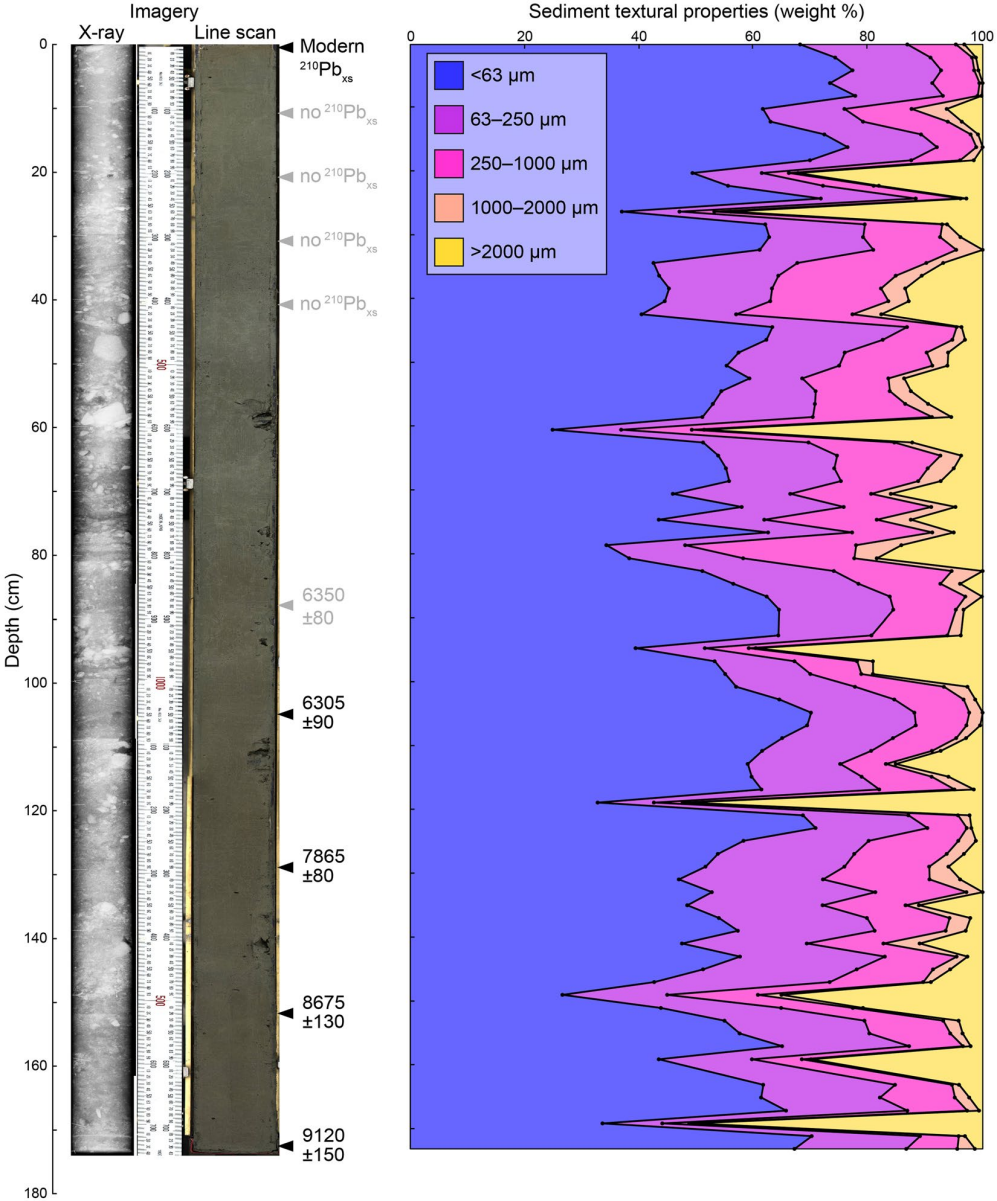
Core description:﻿ ER1116. X-ray and linescan images of ER1116 are shown on a common scale. Black markers show the dated horizons; ^14^C ages are shown in calibrated years BP with errors of ±2*σ*. Samples which returned no excess ^210^Pb are shown in grey. The ^14^C age determination from 88–89 cm is considered unreliable and is also shown in grey (see supplementary material). This figure was created using *Microsoft Excel* 2013 and *Adobe Illustrator CS6*.

The 176 cm long sediment core ER1116 was obtained from the centre of the deep trough in Køge Bugt (64.919 °N, 40.072 °W, and 595 m bsl), approximately 50 km from the present-day ice margin (Fig. 1). Core ER1116 is composed of a single sedimentary facies; a gravelly mud with variable content of coarse clastic material (Fig. 2). Gravel (>2 mm) accounts for up to 52% of the sediment by weight, although average values are much lower (9%). Mean sand and mud (silt + clay) values are 35% and 56% respectively (Fig. 2). X-ray and linescan imagery shows no evidence of sediment disturbance following initial deposition (e.g. erosive contacts or turbidites,Figs. 2 and S2). Sediments are age-constrained by ^210^Pb and ^14^C measurements (Fig. 2). The base of the core is dated to 9120 ±150 cal. years BP. A lack of organic material above 88 cm in the core prevented ^14^C dating of this sediment, and it should be noted that consequently the age model for this part of the core is tentative. ^210^Pb measurements confirm that sediments at the core top are modern.

### Ice-rafted debris

**Figure 3:**
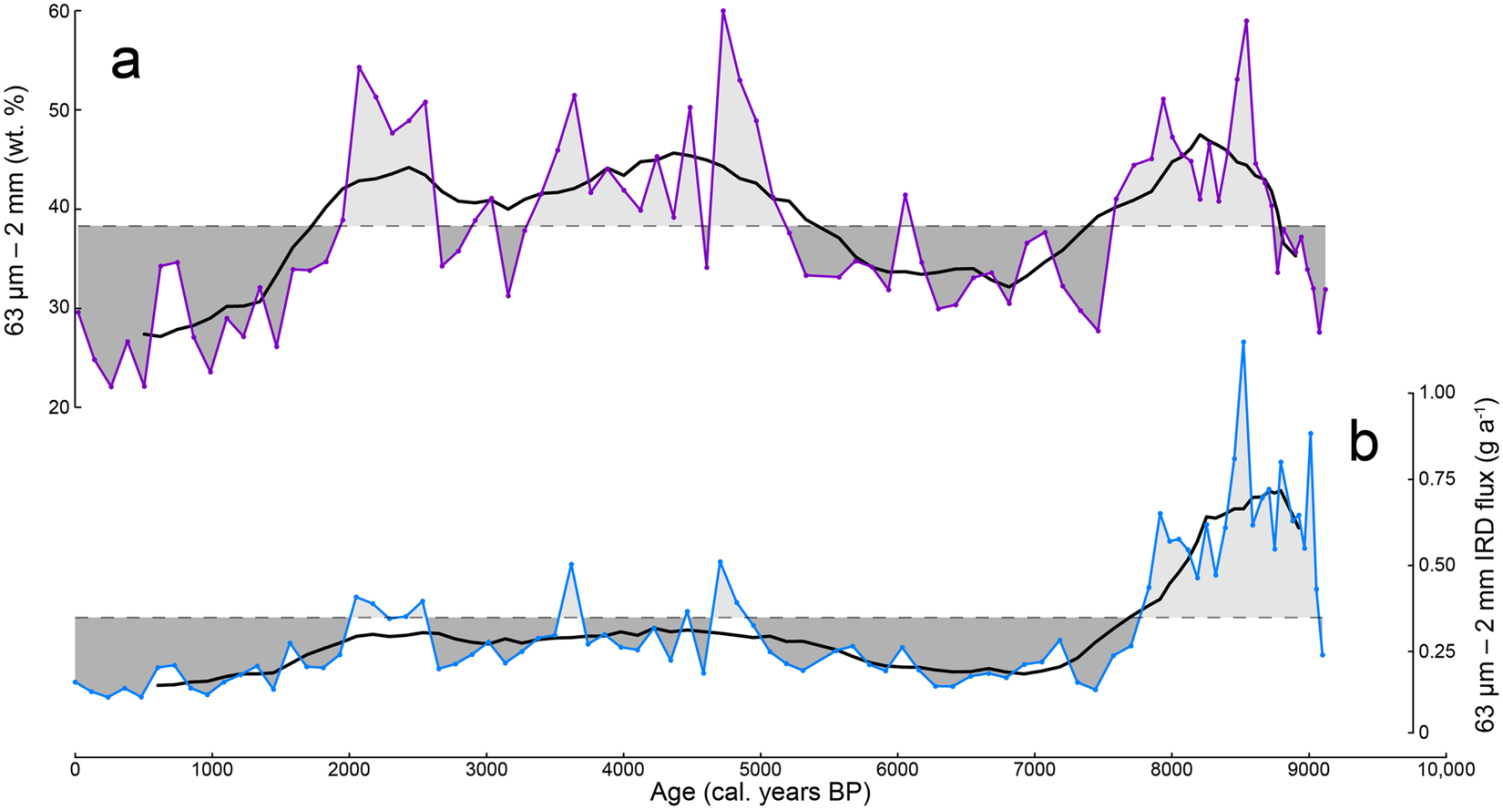
IRD abundance derived from sedimentological analysis. (**a**) The weight of the 63 *μ*m to 2 mm fraction as a percentage of the total sediment weight (excluding clasts >2 mm). (**b**) IRD flux derived from the 63 *μ*m to 2 mm fraction (g yr^−1^). The black lines on both series show the 10-point running mean. Dashed lines show the individual series means. This figure was created using *Microsoft Excel* 2013 and *Adobe Illustrator CS6*.

Ice-rafted debris was quantified using grain size analysis^23^ of sediments in core ER1116 (Fig. 3). The flux of ice-rafted debris (63 *μ*m to 2 mm) was calculated to provide a measure of the variability in iceberg production through time (Fig. 3b). We are confident that coarse sediment (>63 *μ*m) in core ER1116 was deposited exclusively by icebergs calved from the local tidewater glaciers. In some high-latitude environments coarse material can also be transported by sea-ice^24^ and kelp fronds^25^. However, both mechanisms are negligible in Køge Bugt. The absence of sediments in the littoral zone here inhibits the transport of coarse material by sea-ice. Similarly, kelp-plucking does not occur here as shallow marine areas are perpetually abraded by sea-ice and icebergs, and this prevents kelp colonisation. Icebergs from glaciers further north in the region could potentially deliver coarse material to the core site. Non-local icebergs are prevented from entering Køge Bugt by an area of islands and shallow bathymetry to the north of the bay, this obstructs iceberg transit along the inner-shelf^26^. Icebergs further offshore are advected past the bay as they are entrained in fast, southerly-flowing ocean currents (Fig. 1).

Ice-rafted debris data are inherently noisy because of the stochastic nature of iceberg sedimentation. Consequently, minor or short-lived variations should be interpreted cautiously. However, at a Holocene timescale the data reveal an important result; the record shows that ice-rafted debris sedimentation occurred continuously in Køge Bugt throughout the last 9100 years (Fig. 3).

### Foraminifera

**Figure 4:**
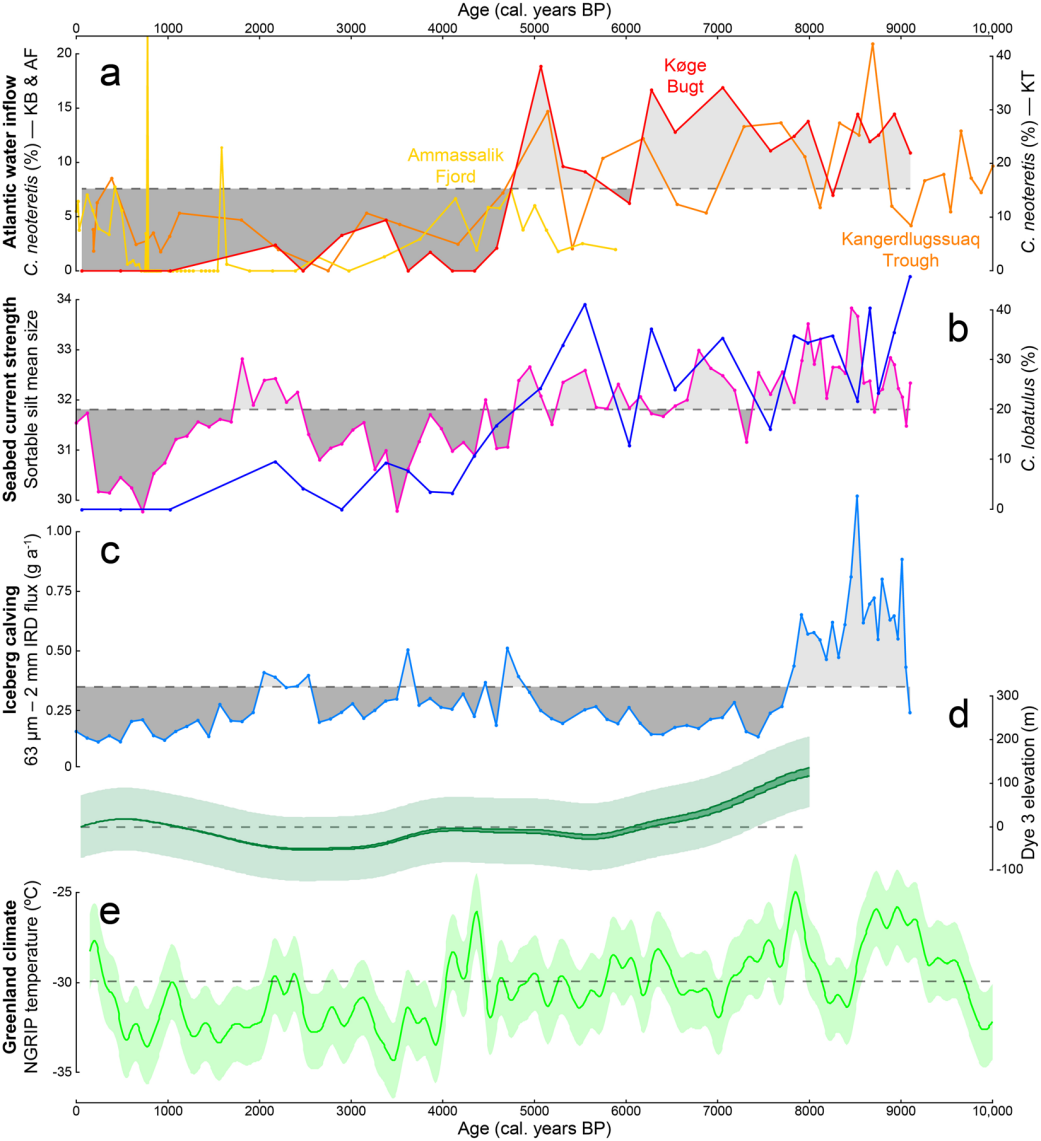
Holocene ocean, climate, and ice sheet elevation records from Køge Bugt. (**a**)* Cassidulina neoteretis* % abundance from sites in southeast Greenland. Red–Køge Bugt (this study); Orange–Kangerdlugssuaq Trough^45,46^; Yellow–Ammassalik Fjord^45^. Data from Køge Bugt (KB) and Ammassalik Fjord (AF) are plotted against the left axis, data from Kangerdlugssuaq Trough (KT) are plotted against the right axis. (**b**) Sortable silt mean grain size (magenta), this is overlain with the % abundance of *Cibicides lobatulus* (blue). (**c**) Flux of IRD from the 63 *μ*m to 2 mm fraction %. (**d**) Dye 3 elevation with 1*σ* uncertainty envelope^52^. (**e**) NGRIP temperature history with 2*σ* uncertainty envelope^68^. Dashed lines show the means of individual records. This figure was created using *Microsoft Excel* 2013 and *Adobe Illustrator CS6*.

The record of foraminiferal assemblage change provides information about oceanographic conditions in Køge Bugt through most of the Holocene. Here, we focus on the abundance of selected indicator species to highlight oceanographic changes (Fig. 4). Full results and detailed interpretations are provided in the accompanying supplementary material. We use the percentage abundance of *Cassidulina neoteretis*, a species known to thrive in warm, saline, Atlantic water masses^27–29^, as a proxy for Irminger Current incursion into Køge Bugt. The abundance of *Cibicides lobatulus* provides a proxy for current strength at the seabed as it is widely associated with coarse sediments and high-energy environments^28–33^.

The results show a similar pattern of *Cassidulina neoteretis* and *Cibicides lobatulus* abundance through the sediment core. High abundances of both species in the early-Holocene suggest that Køge Bugt was characterised by warm, saline waters and high hydrodynamic energy levels at the seabed during this interval (Fig. 4). A marked reduction in the abundance of both species occurred at around 5000 years BP, with low values through the remainder of the Holocene. This indicates that the late-Holocene in Køge Bugt was characterised by low current strengths at the seabed and cold oceanographic conditions.

### Sortable silt

Measuring the mean grain size of the sortable silt fraction (10–63 *μ*m^34^) can provide a record of the relative changes in hydrodynamic energy at the seabed^35^. This technique has been widely used in deep-sea and continental slope environments, in these settings the variability reflects changes in the selective deposition of silt grains^36^. A reduction in current speed allows finer particles to settle out of suspension during long-distance transport; this results in a smaller mean sortable silt grain size at distal sites^35^. In contrast, at the core location in Køge Bugt, sediment is primarily deposited *in situ* by iceberg-rafting; this deposits sediment across the grain size spectrum (from clay to boulders). We argue that silt sorting in these settings primarily occurs after sediment deposition through the preferential erosion of fine- grained sediment (winnowing).

Current-sorting of marine muds has only been sparsely investigated in glaciomarine environments^37^ as the deposition of large ice-rafted clasts can potentially interfere with the current-sorting signal by shielding underlying sediment from winnowing^38–41^. However, deposition of ice-rafted debris is only problematic when sedimentation rates are rapid (>1 cm yr^−1^); in areas with lower sedimentation rates well-sorted sediments can be produced under just a few days of fast-flow conditions (i.e. 10 cm s^−1^) a year^35^. Our results suggest that increases in the mean grain size of the sortable silt fraction are primarily caused by increased current flow at the seabed; the degree of sorting of the silt fraction (*σ*) increases concurrently with the mean grain size (Fig. S8).

The sortable silt data exhibit substantial variability throughout the last 9100 years (Fig. 4b). The sortable silt grain size values are highest from 9100 to 5000 years BP, suggesting a period of enhanced current circulation at the seabed. This is corroborated by the high abundance of *Cibicides lobatulus* during this interval (Fig. 4b). A second notable increase in the sortable silt grain size occurs from ~2500 to 1500 years BP, similarly, this is accompanied by an increase in silt sorting (*σ*) and high abundances of *Cibicides lobatulus* (Figs. 4b and S8b).

## Discussion

Modern temperatures at depth in Køge Bugt are around 4 °C (Fig. S1), however, the warm water indicator species *Cassidulina neoteretis* is absent from modern sediments (Fig. 4a). The high abundance of *Cassidulina neoteretis* in the early-Holocene suggests that deep ocean temperatures at this time were at least as warm as the present-day (i.e. ~4 °C). The Irminger Current is the only source of warm, saline water masses in southeast Greenland^42^; we postulate that subsurface waters in Køge Bugt were well-ventilated by the Irminger Current during the early-Holocene. This interpretation is supported by high sortable silt grain sizes and the abundance of *Cibicides lobatulus* during this interval (Fig. 4b). Foraminifera and sortable silt data suggest a significant shift towards colder oceanographic conditions and reduced current activity in Køge Bugt at around 5000 years BP (Fig. 4). The cooling trend continued through the late-Holocene, culminating at around 1000 years BP, when Køge Bugt was characterised by cold, Polar conditions (Fig. 4); this was probably linked to global-scale climatic and oceanic cooling at this time^43,44^. Our new oceanographic reconstructions are in good agreement with existing data from two areas further to the north in southeast Greenland; Ammassalik Trough^45^ and Kangerdlugssuaq Trough^46^ (Fig. 4a). The regional coherence of ocean reconstructions throughout the Holocene suggests that the data reflect large-scale changes in the structure and strength of the Subpolar Gyre.

The delivery of iceberg-rafted sediments to the core site is controlled by the interplay between glaciological, climatic, and oceanic conditions. Unravelling the significance of episodes of ice-rafted debris requires an understanding of a range of environmental conditions. We interpret the ice-rafted debris record in the context of new and existing independent proxy records from the region (Fig. 4). However, it is important to note that, whilst we endeavour to uncover the most plausible scenarios, the processes controlling ice-rafted debris sedimentation are complicated and our interpretations are tentative.

Elevated delivery of iceberg-rafted debris occurred from 9000 to 7800 years BP, this overlaps with an interval of ice sheet lowering at the Dye 3 site, which is located within the Køge Bugt glacial catchment (Fig. 4d). This episode of iceberg-rafted debris probably reflects a period of high glacial discharge as the interior of the Greenland Ice Sheet adjusted to climate warming after the Last Glacial Maximum through ice margin retreat and ice sheet thinning^47,48^. Alternatively, this episode may be directly attributable to the warm oceanic conditions in Køge Bugt during the early-Holocene (Fig. 4a). Under this scenario we envisage that the increase in ice-rafted debris was caused by an intensification of glacier calving, triggered by submarine melting at the glacier terminus^5,6^. This was followed by an interval of reduced ice-rafted debris input from 7800 to 5000 years BP (Fig. 4c). This coincided with the peak of the Holocene Thermal Maximum^43,49,50^ when regional ocean (Fig. 4a) and climate conditions (Fig. 4e) were at least as warm as the 20th Century. The reduction in ice-rafted debris during this period probably resulted from increased melt-out of sediment from icebergs before it could reach the core site in the outer-part of Køge Bugt. A modest increase in ice-rafted debris accumulation occurred from 5000 to 2000 years BP. This period was characterised by the establishment of the Neoglacial; glaciers around Greenland^50^, and the wider North Atlantic region^51^, advanced in response to substantial oceanic^44^ and atmospheric^43^ cooling during this interval (Fig. 4). We interpret that cooler conditions reduced iceberg melting during transit, this allowed more debris-rich icebergs to reach the core site. From 2000 years BP onwards there was a substantial reduction in ice-rafted debris (Fig. 4c), this occurred during the coldest conditions of the Holocene. We suggest that ice-rafting of sediment to the site of ER1116 was restricted by ice conditions in Køge Bugt during this interval. Longer periods of sea-ice cover and increased cohesion of glacial mélange likely restricted the transit of debris-laden icebergs to the core site.

**Figure 5:**
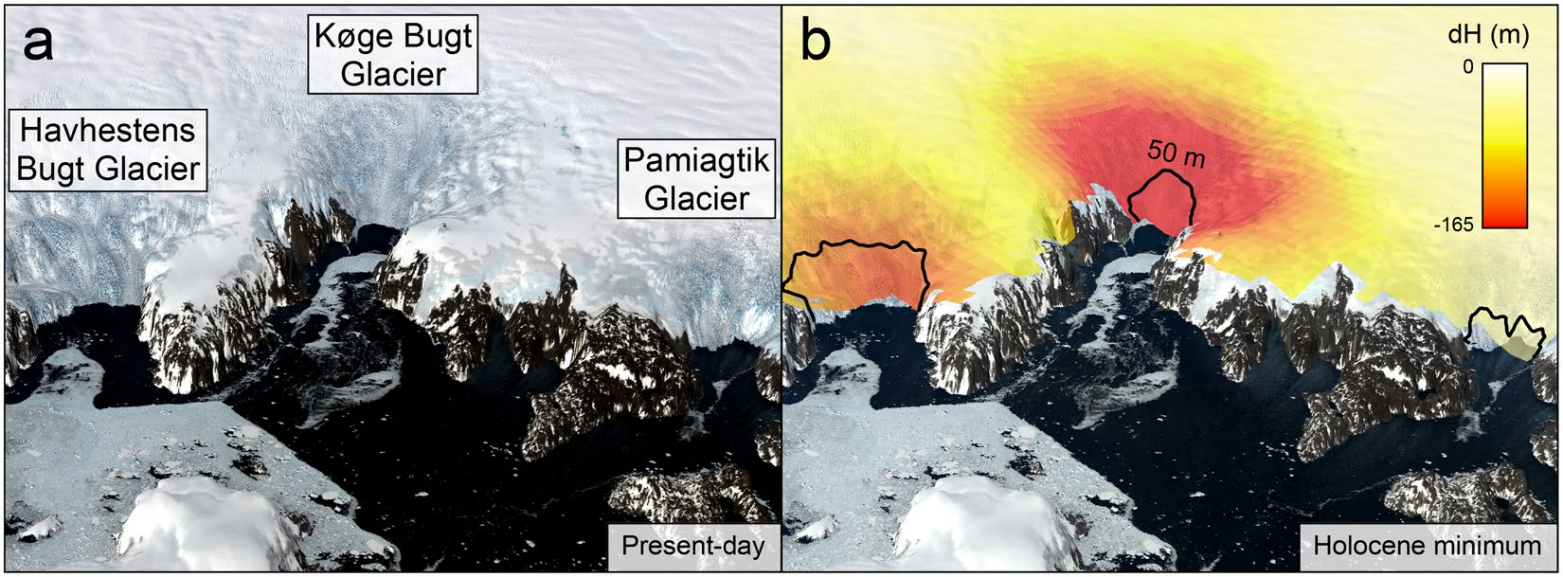
Visualisation of Holocene glaciological changes in Køge Bugt (5x vertical exaggeration). The scene looks northwest over Jens Munks Ø towards the three major outlet glaciers in Køge Bugt. (**a**) Modern glacier configuration. Landsat 8 imagery^69^ is projected onto GIMP surface elevation data^67^ (2011 census date). (**b**) Minimum Holocene glacier extent and mass loss in Køge Bugt. Modern Landsat imagery is draped on the ice surface calculated for the minimum Holocene extent glacier surface (see Methods). The black line marks the position of the 50 m bed contour beneath the glaciers in Køge Bugt^15^. The colour scale shows thinning relative to the present-day ice surface (2012). This figure was created using *ArcMap* 10.1, *ArcScene* 10.1, and *Adobe Illustrator CS6*.

The ice-rafted debris record displays variability through the Holocene that reflects changes in both iceberg production and oceanographic conditions. However, when examining glacial behaviour, the most significant observation is that ice-rafted debris is present continuously throughout core ER1116 (Fig. 3). This demonstrates that the large outlet glaciers in Køge Bugt have remained in tidewater settings for the last 9100 years. Subglacial troughs extend to a maximum of 6 km inland from the present-day glacier fronts (Fig. 1). Consequently, glaciers in Køge Bugt have not retreated more than 6 km in the last 9100 years. This was despite regional ocean temperatures (Fig. 4a) and climatic conditions^50^ that were at least as warm as the present-day. The minimal retreat of glaciers in Køge Bugt is consistent with the ice sheet elevation record derived from Dye 3^52^. Surface lowering had ceased here by approximately 6000 years BP, and was stable through the remainder of the Holocene as glaciers in Køge Bugt remained in tidewater configurations (Fig. 4d). We calculate the changes in glacier geometry for a maximum Holocene retreat scenario using scaling factors derived from modern glacier changes^53^ in Køge Bugt. At their theoretical most-retreated position (6 km inland of the present-day margin), glaciers here lost an estimated 127 Gt (141 km^3^) relative to the present-day configuration (Fig. 5). This upper-bound on Holocene mass loss compares with 21^*st*^ Century mass loss of 36 Gt (40 km^3^) for these glaciers^12^. Maximum Holocene glacier retreat of 6 km (relative to the present-day) is broadly comparable to changes during the 20^*th*^ and 21^*st*^ Centuries; between 1931 and 2012 the central outlet, Køge Bugt Glacier, retreated 5 km^10,53^.

The minimal retreat of glaciers in Køge Bugt during the Holocene contrasts with other areas of southeast Greenland where large glaciers are thought to have retreated onto land during the Holocene Thermal Maximum^46,54^. Small land-terminating glacier systems in the region also retreated substantially (3–5 km) during the peak of the Holocene Thermal Maximum^55^. These new results constrain the frontal position of glaciers in Køge Bugt, but do not provide a direct record of the calving flux, which likely varied through the Holocene. Nevertheless, our results demonstrate that the glaciers here are relatively stable, even when subjected to large external forcings. We attribute this behaviour to their unique bathymetry and subglacial topography; the bed slopes upwards rapidly inland from the present-day margin. This configuration allows the glaciers to adjust to new stable positions with only a minor retreat^56^. The steep surface profile of the ice sheet in Køge Bugt, which reflects the subglacial topography (Fig. 1), also enhances the stability of these glaciers as it makes them relatively insensitive to changes in equilibrium line altitude. Additionally, very high accumulation rates in the area^17,18^ may also have played a role in buffering these glaciers from warm oceanic and climatic conditions in the early-Holocene. We conclude that the stability of glaciers in Køge Bugt through the Holocene is primarily attributable to their steep topographic setting.

This study highlights that the physical setting of glaciers in Køge Bugt has controlled their response to external forcing. This underlines the importance of mapping the subglacial topography and bathymetry of glacier systems in order to understand their past, present, and future behaviour. The first decade of the 21st Century was marked by significant mass loss from glaciers in Køge Bugt. However, we suggest that, unlike most other glaciers in southeast Greenland, glaciers in Køge Bugt will remain in configurations similar to the present-day into the near-future, despite the predicted continuation of atmospheric warming^57^. Finally, these results provide a baseline against which to assess the magnitude of glacier change in Køge Bugt. If glaciers in Køge Bugt continue to retreat, and become land-terminating, this will be unprecedented in at least the last 9100 years.

## Methods

We analysed marine sediments to reconstruct the Holocene glacial and oceanographic history of Køge Bugt. A Rumohr corer with 80 mm diameter liner was used to obtain the 176 cm sediment core. A core catcher device was not used; this ensured that surface sediments were not disturbed during coring.

### Core description and non-destructive analysis

The core was split lengthways prior to analysis. Half of the core was subjected to non-destructive analysis using X-ray imaging (Copenhagen, DK) and line scanning (NIOZ, Den Hoorn, NL), this was then archived. X-ray imaging was used to examine sedimentary structures in ER1116. Individual high-resolution (0.09 mm px^−1^) X-ray images were obtained at ~20 cm intervals, these were spliced into a single composite image using functions in *Adobe Photoshop CS6*.

### Chronological constraint

The chronology for ER1116 was built from five ^210^Pb and five ^14^C age determinations. Bulk sediment samples (~10 cm^3^) for ^210^Pb dating were taken at ten centimetre intervals from the uppermost 50 cm of ER1116 (Fig. S3). Radiocarbon dates were obtained from samples of >1000 foraminifera tests. Limited foraminifera content in the upper- 88 cm in the core prevented ^14^C dating of this sediment. Material for ^14^C dating was analysed at Aarhus University, Denmark (n = 1) and the Beta Analytic laboratory, USA (n = 4). Radiocarbon dates were calibrated with *CALIB* 7.0 (calib.qub.ac.uk/calib/) using the Marine13 calibration curve^58^. A marine reservoir correction of 400 years (ΔR = 0) was applied to all ^14^C dates^59^, as the benthic foraminiferal assemblages used for dating indicate an Atlantic water origin.

### Sediment grain size analysis

The grain size distribution of sediment in ER1116 was determined by wet-sieving and laser diffraction analysis. Material was sampled from 10 cm^3^ of sediment, representing a 1 cm core slice, at 2 cm intervals, from the full-length of ER1116. Water content was determined prior to sieving by freeze-drying and weighing. Sediment was wet-sieved through a stack of *Retsche* sieves (2 mm, 1 mm, 250 *μ*m, and 63 *μ*m). The >2 mm fraction was excluded from the percentage calculations to remove large, anomalous peaks caused by the presence of large individual clasts.

Laser diffraction analysis was used to determine the size distribution of fine-grained sediments (0.3–63 *μ*m^34^). Analysis was undertaken with a *Malvern Mastersizer* 3000. Samples were pretreated with tetrasodium pyrophosphate (0.01 M Na_4_P_2_O_7_ · 10 H_2_O) and submerged in an ultrasound bath for two minutes to disaggregate particles prior to measurement^37^. Analysis of a sample typically takes ~10 seconds and produces measurements with errors of less than ±1%.

### Foraminiferal assemblage analysis

Foraminiferal assemblage analysis was undertaken every 8 cm in ER1116. Additional subsamples were collected at 4 cm intervals in areas of the core where rapid shifts in species assemblage occur. Foraminifera tests were obtained from 1 cm slices of core material (~9 cm^3^). Subsamples were wet-sieved and the 100 *μ*m to 1 mm size fraction was selected for analysis^60^. The 63–100 *μ*m size fraction was also checked for foraminifera content. Foraminifera tests were concentrated by flotation using heavy liquid (C_2_Cl_4_, 1.62 g cm^−3^) and then dry picked from a graticuled tray using a stereo microscope. Both planktonic and benthic foraminifera tests were picked. Where possible at least 300 individual benthic tests were picked, this enables statistically robust analysis^28,61^. In many intervals foraminifera tests were sparse. Samples with fewer foraminifera provide less-robust data. Nonetheless, these yield valuable information about environmental conditions. The percentage abundance of individual species was calculated against the total of agglutinated or calcareous tests in each sample.

### Holocene glacier elevation and mass loss changes

Mass loss from glaciers in Køge Bugt was calculated by establishing a linear relationship between modern glacier thinning (d*h*_2003–2012_^53^) and retreat (d*r*_2003–2012_). This was then extrapolated inland to the 50 m bed contour (d*r*_2012–HolMin_), the minimum possible extent of glaciers here during the Holocene (Fig. 5b), to obtain the upper-bound of Holocene thinning relative to 2012 (d*h*_2012–HolMin_). The 50 m contour was used to account for isostatic depression of the crust in the early-Holocene. Field observations^62^ and a compilation of relative sea-level limits^63^ demonstrate that the maximum isostatic depression in the Køge Bugt area was between 20 and 40 m. We conservatively use the 50 m contour for all calculations. Modern glacier retreat was calculated from Landsat 7 imagery with a modified centre-line method^64^ using the mean of three flow lines orientated perpendicular to the glacier front. The relationship between glacier retreat and thinning was established from the central outlet glacier (Køge Bugt Glacier) as this has experienced the largest rates of modern retreat and thinning. This glacier also has a simple margin geometry. The volume of ice loss during minimum Holocene glacier extent was calculated for each grid cell (1 km × 1 km) in the Køge Bugt glacial basin (13_a)^53^ using:

1 $${\rm{d}}{h}_{2012-{\rm{HolMin}}}={\rm{d}}{h}_{2003-2012}\cdot (\frac{{\rm{d}}{r}_{2012-{\rm{HolMin}}}}{{\rm{d}}{r}_{2003-2012}})$$

Individual grid values were then summed to obtain an estimate of volume loss from the glaciological basin (km^3^) and multiplied by 0.9 to obtain an estimate of ice mass loss (Gt). This approach assumes that the surrounding outlet glaciers in Køge Bugt behaved synchronously. It also relies on the assumption that glacier thinning between 2003 and 2012 had equilibrated to changes in margin position, and that the relationship between calving front position and thinning has remained constant throughout the Holocene. This method provides a first-order approximation of maximum ice loss from glaciers in Køge Bugt over the last 9100 years.

## Electronic supplementary material

Supplementary material
