# Supplementary material for: Minimal Holocene retreat of large tidewater glaciers in Køge Bugt, southeast Greenland
Source: Sci Rep. 2017 Sep 26;7:12330. doi: 10.1038/s41598-017-12018-x (PMC5615072; doi:10.1038/s41598-017-12018-x)
Supplement: Supplementary file 1 — Supplementary material [file 41598_2017_12018_MOESM1_ESM.pdf]

Supplementary material: Minimal Holocene retreat of large tidewater glaciers in Køge Bugt, southeast Greenland

Laurence M. Dyke[1,*], Camilla S. Andresen[1], Marit-Solveig Seidenkrantz[2], Anna L. C. Hughes[3], John F. Hiemstra[4], Tavi Murray[4], Anders A. Bjørk[5], David A. Sutherland[6], and Flor Vermassen[1]

[1]Geological Survey of Denmark and Greenland, Department of Glaciology and Climate, Øster Voldgade 10, DK-1350 København K, Denmark.
[2]Centre for Past Climate Studies, Department of Geoscience, Aarhus University, Høegh-Guldbergs Gade 2, DK-8000 Aarhus C, Denmark.
[3]Department of Earth Science, University of Bergen and Bjerknes Centre for Climate Research, Allégaten 41, N-5007 Bergen, Norway.
[4]Glaciology Group, Swansea University, Singleton Park, Swansea, SA2 8PP, UK.
[5]Centre for GeoGenetics, Natural History Museum of Denmark, University of Copenhagen, Øster Voldgade 5–7, DK-1350 København K, Denmark.
[6]Department of Geological Sciences, 1272 University of Oregon, Eugene, OR 97403-1272, USA.
[*]Correspondence and requests for materials should be addressed to L.M.D. (email: lad@geus.dk).

ABSTRACT

This document contains supplementary material for 'Minimal Holocene retreat of large tidewater glaciers in Køge Bugt, southeast Greenland'.

Study area: Køge Bugt

This section contains additional information about Køge Bugt, the study area of this investigation. The terrain around Køge Bugt is relatively subdued; the highest nunataks, 10s of kilometres inland, are ∼1300 m asl[1]. The Greenland Ice Sheet has a relatively steep profile in this area; it rises to 2550 m asl at the ice divide, just 160 km inland from Køge Bugt. The northern boundary of the bay is characterised by several low-lying peninsulas and islands (Fig. S1). Jens Munks Ø forms the southern edge of the bay; this large island stands 883 m asl[2] and is almost entirely glaciated by an independent ice cap (Fig. S1). The ice-free areas of Køge Bugt bear the signature of intense glacial erosion and are almost entirely devoid of sediment and vegetation cover. Satellite imagery (Landsat 8 scenes, https://earthexplorer.usgs.gov/) and aerial photos[3] reveal the presence of minor glacial trimlines around the northern outlet glacier ('Pamiagtik Glacier'[4]), and small moraines close to the present-day margin in land-terminating areas. These features are assumed to delineate the Little Ice Age maximum extent, similar to elsewhere in southeast Greenland[5–7].

The geology of Køge Bugt is predominantly composed of reworked Archaean gneisses of the Nagssugtoqidian Mobile Belt; these are extremely resistant to erosion. Millimetre-scale striations and glacially polished surfaces are preserved in gneiss surfaces that most likely became deglaciated at the start of the Holocene[8,9].

There are very few existing oceanographic measurements from the area around Køge Bugt; the closest CTD profiles are from >50 km to the east of the bay, data from these are shown in Fig. S1c. The only direct oceanographic measurements from within Køge Bugt are temperature profiles obtained with marine mammal-borne sensors[10–12]. There are more than 200 individual seal dive profiles from Køge Bugt and the adjacent continental shelf. However, most are shallow (<200 m) and thus reflect the cold, Polar water masses near the surface. A handful of deeper temperature profiles (>300 m) provide a more complete picture of oceanographic conditions in the area (Fig. S1b).

Sediment coring

The sediment core ER1116 was collected from Køge Bugt (64.919 °N, 40.072 °W, and 595 m bsl) during the SEDIMICE project field campaign in August 2011. Coring was undertaken from the R/V Þytur using a Rumohr coring system[17]. Core

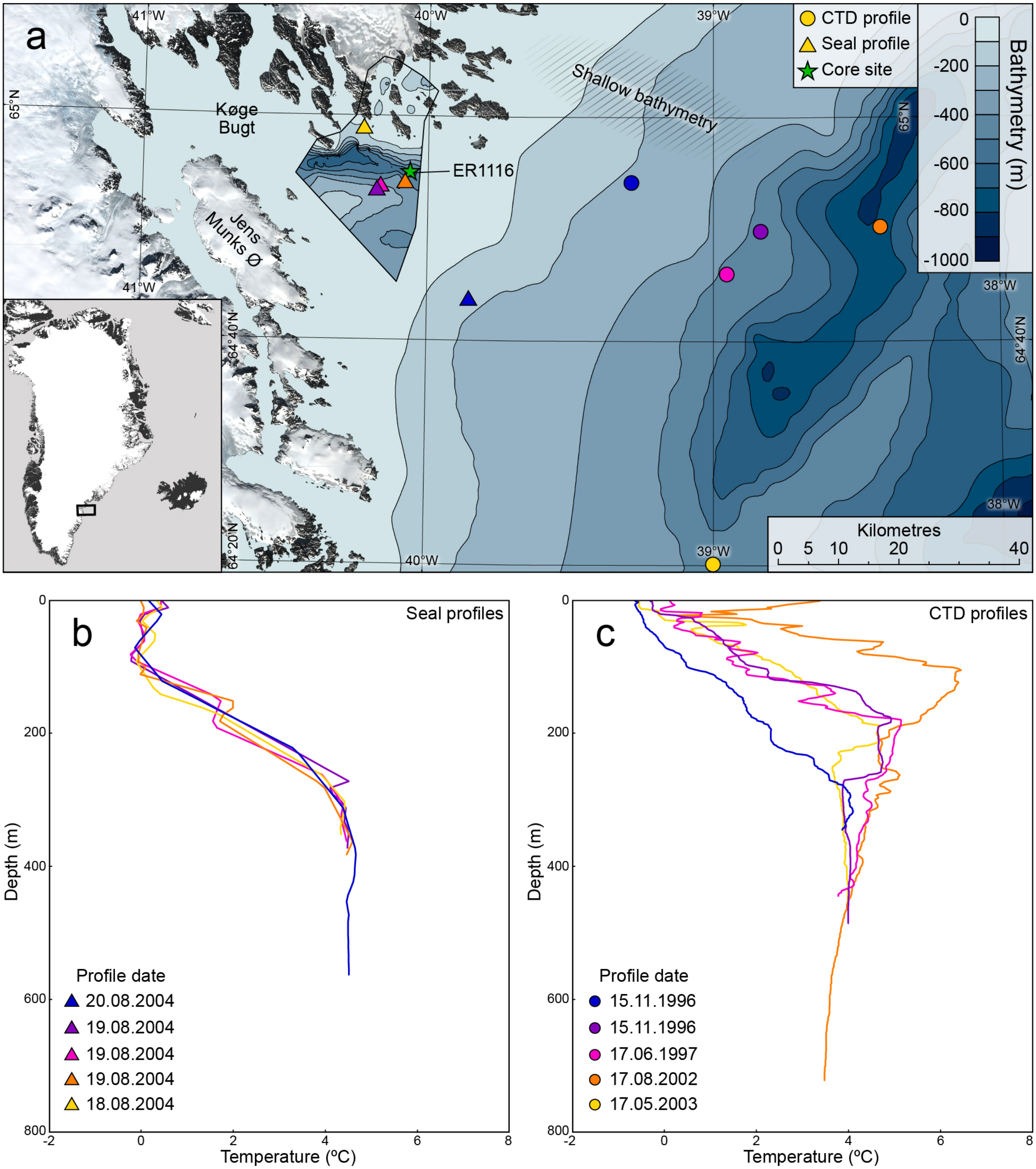

Figure S1. The oceanography of the Køge Bugt area. (a) 1:900,000 map showing the bathymetry of the continental shelf[13] and the locations of CTD[14,15] and seal dive[12] profiles. Symbols mark the locations of the CTD profiles (circles) and seal dive profiles (triangles). The location of core ER1116 is marked by a star. The inset map shows the location and extent of the main figure. Background imagery are Landsat 8 scenes[16]. (b) Temperature profiles from deep (>300 m) seal dives in Køge Bugt[12]. (c) Temperature profiles from CTD stations; these were produced with data from the World Oceanographic Database 2013 (WOD13) and the International Council for the Exploration of the Sea (ICES)[14,15]. This figure was created using *ArcMap 10.1* and *Adobe Illustrator CS6*.

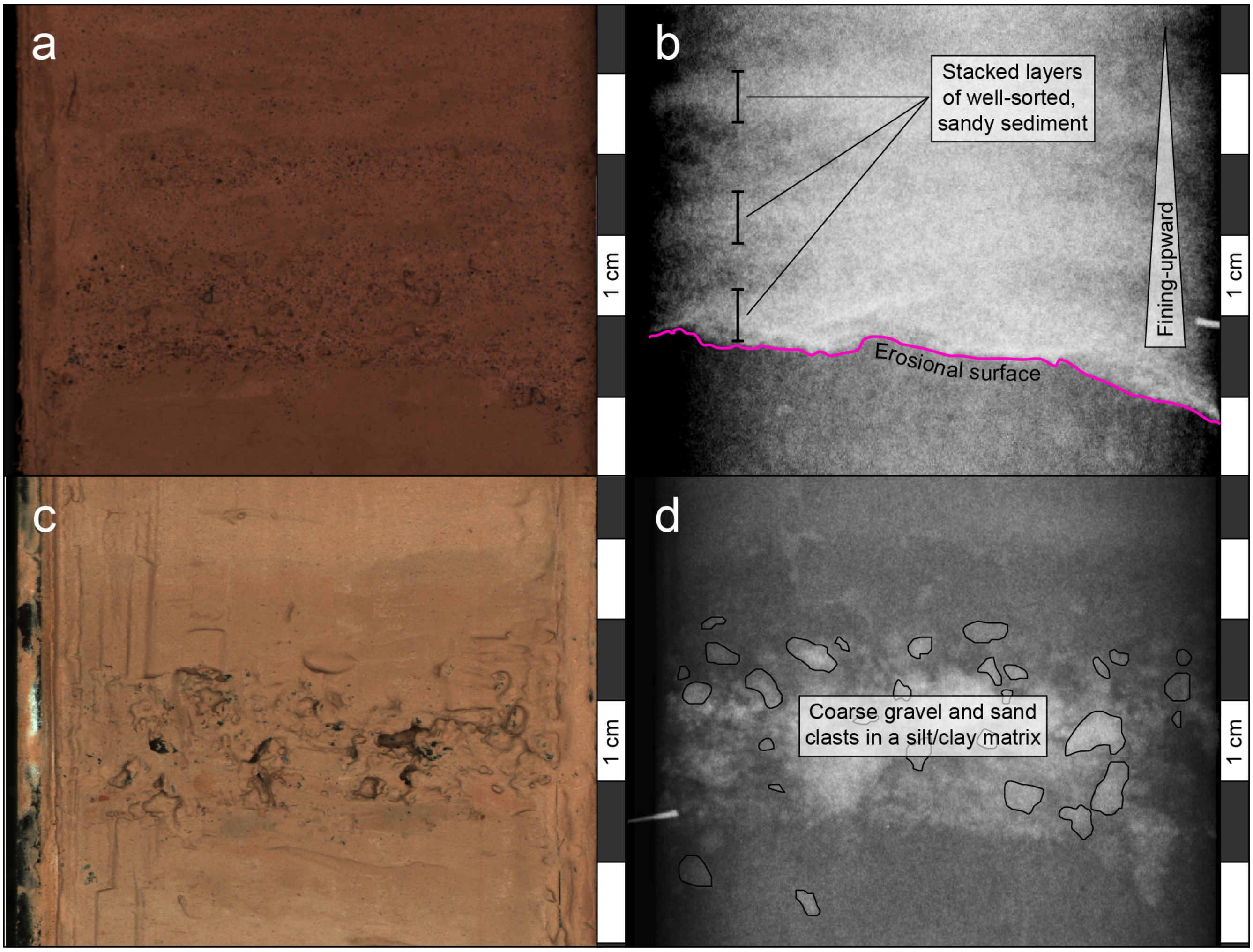

Figure S2. Distinguishing between turbidites and layers with high ice-rafted debris content. (a) and (b) Line scan and X-ray imagery showing an example of a stacked turbidite sequence (core POR13-05, Upernavik, northwest Greenland). The turbidite is characterised by a sharp, undulating erosional basal surface, a general fining-upward trend, and stacked layers of sandy/gravelly sediment. (c) and (d) Line scan and X-ray imagery of a layer of high ice-rafted debris content (core POR13-16, Upernavik, northwest Greenland). This layer is characterised by diffuse upper- and lower-boundaries, numerous coarse sand and pebble-sized clasts, and a lack of internal layering structures. This figure was created using *Adobe Illustrator CS6*.

ER1116 was immediately sealed and stored vertically for transport to the core facility at The Geological Survey of Denmark and Greenland (Copenhagen, DK).

Examination of sediment for disturbance events

Marine sediments in high-latitude environments are frequently disturbed by iceberg ploughing and by mass-wasting events, such as debris flows and turbidity currents[18,19]. It is important to identify these events when establishing the stratigraphy of a sediment core. In this study the effect of iceberg ploughing was mitigated by coring from the centre of the deep trough in Køge Bugt, this is thought to be well-below the keel-depth of even the largest icebergs here. We used line scan and X-ray imagery to examine core ER1116 for the presence of turbidites and to distinguish these from layers rich in ice-rafted debris on the basis of morphological and sedimentological characteristics (Fig. S2). Core ER1116 shows no evidence of large turbidites, although we consider it possible that minor, millimetre-scale turbidites may be present.

Dating constraints and age model

Dating sediments from glacier proximal environments is extremely challenging; harsh conditions restrict marine life and ^{14}C dating is not always possible. The scarcity of organic material is compounded by high sedimentation rates in these settings[20]. Sediments in ER1116 were age-constrained by five ^{14}C dates and five ^{210}Pb age determinations. Radiocarbon dates were obtained from benthic calcareous foraminifera, in the upper-half of the core these are sparse and it was necessary to combine planktonic and benthic foraminifera with bivalve shell fragments to obtain sufficient material for ^{14}C dating.

Table 1. ^{210}Pb measurements from ER1116 (64.919 °N, 40.072 °W, and 595 m bsl). Analysis was undertaken on freeze-dried sediments at the University of Bordeaux using standard procedures[21].

Depth	Material dated	Sediment weight (g)	^{210}Pb mBq g^{-1}	$^{210}Pb_{xs}$
0–1 cm	Bulk sediment	9.268	202 ±7	193 ±7
10–11 cm	Bulk sediment	9.359	11 ±3	3 ±3
20–21 cm	Bulk sediment	9.633	8 ±3	−1 ±3
30–31 cm	Bulk sediment	9.629	7 ±2	−1 ±3
40–41 cm	Bulk sediment	9.789	7 ±2	−2 ±2

The base of ER1116 (172–174 cm) was dated to 9120 ±150 cal. years BP (Table 2). The remaining ^{14}C age determinations exhibit a simple age-depth relationship, with the exception of the date from 88–89 cm (Fig. S3). Sediment from this interval was very sparse in calcareous benthic foraminifera tests. It was necessary to supplement the benthic tests with planktonic foraminifera and shell fragments from an unidentified species of bivalve to provide enough organic material to measure the $^{12}C/^{14}C$ ratio. Detailed inspection of the supplementary bivalve shell during preparation for ^{14}C analysis revealed a dull lustre and 'chalky' texture; this suggests it may have been composed of replaced carbon (C. Patrick, personal communication, 2014). This interpretation is supported by the anomalously old ^{14}C age; this date is subsequently excluded from the age model (Fig. S3).

Results from lead-210 dating (Table 1) demonstrate that the core top is composed of modern material; this provides a tie-point for the age model here (Fig. S3). Intervals below this contained no unsupported (excess) ^{210}Pb (Table 1). The lack of unsupported ^{210}Pb in these samples is consistent with the modelled age of these sediments (Fig. S3).

Age model

The age-depth relationship is established using simple linear interpolation between chronostratigraphic tie-points (Fig. S3). Sedimentation rates were highest in the early-Holocene ($\sim$46 cm kyr^{-1}) and dropped to $\sim$15 cm kyr^{-1} by 8000 years BP. A single sedimentation rate of $\sim$16 cm kyr^{-1} is calculated for the entire period from 6300 years BP to the present. It should be noted that this may not accurately reflect sedimentation rates over shorter timescales within this interval.

The age-model is relatively coarse; the absence of organic material in late-Holocene sediments means chronological uncertainties in this interval are large (Fig. S3). Nonetheless, the ^{14}C and ^{210}Pb results indicate that the sediments cover the last 9100 years of sedimentation in Køge Bugt. This is supported by linescan and X-ray imagery which shows no evidence for

Table 2. Radiocarbon age determinations from sediment core ER1116 (64.919 °N, 40.072 °W, and 595 m bsl). Calibrated using CALIB 7.0[22], the Marine13 calibration curve[23], and a marine reservoir correction of 400 years, $\Delta R = 0$[24,25]. Calibrated ages show the median of the 2σ range with 2σ uncertainties. The material from 88–89 cm was composed of benthic and planktonic foraminifera tests in addition to shell fragments from an unidentified species of bivalve.

Depth	Material dated	Lab ID	^{14}C age (yr)	2σ cal. age (yr BP)
88–89 cm	Foram. tests and bivalve fragments	Beta390352	5940 ±30	6350 ±80
104–105 cm	Benthic foram. tests	Beta390353	5890 ±40	6305 ±90
128–129 cm	Benthic foram. tests	Beta387758	7400 ±30	7865 ±80
152–153 cm	Benthic foram. tests	Beta387759	8180 ±30	8675 ±130
172–174 cm	Benthic foram. tests	AAR18323	8480 ±55	9120 ±150

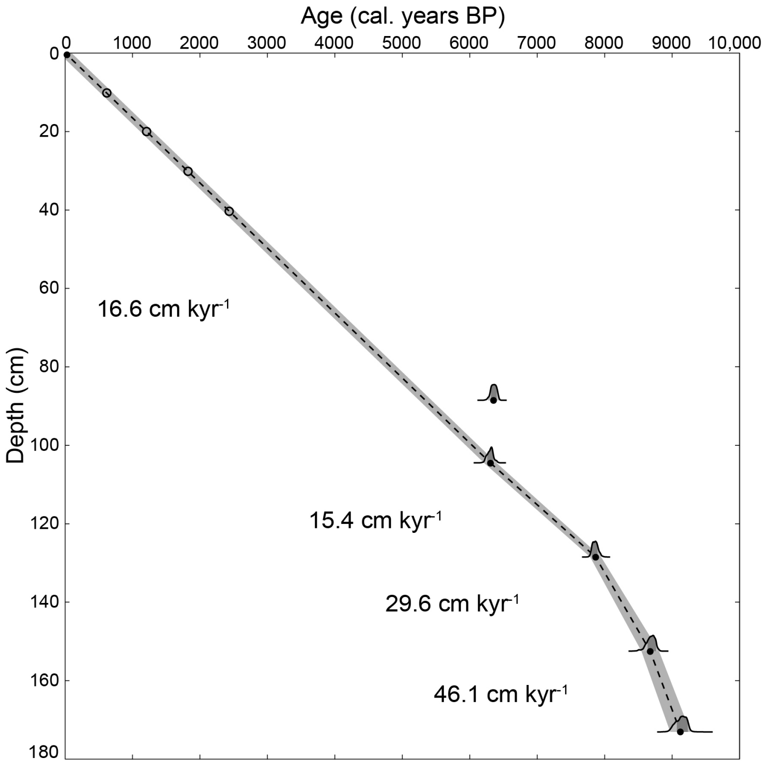

Figure S3. ER1116 age model based on five ^{210}Pb age determinations (Table 1) and five ^{14}C dates (Table 2). Hollow circles show locations of ^{210}Pb samples that returned no excess ^{210}Pb, these are not chronological tie-points. However, these results are consistent with the age model. The ^{14}C date from 88–89 cm was excluded from the age model (see text). Calibrated probability curves for each ^{14}C age are shown. This figure was created using *CALIB 7.0* (calib.qub.ac.uk/calib/) and *Adobe Illustrator CS6*.

sediment disturbance or hiatuses (Fig. S2).

Foraminifera assemblage analysis

Detailed results from foraminifera assemblage analysis are presented here (Figs. S4, S6, S5, and S7). Foraminiferal assemblage analysis was undertaken every 8 cm in ER1116. Additional subsamples were collected at 4 cm intervals in areas of the core where rapid shifts in species assemblage occur. Foraminifera tests were collected from 1 cm slices of core material, consequently, the species assemblage in an individual sample represents an average of conditions during the period of sediment accumulation. Subsamples were wet-sieved, the 100 μm to 1 mm size fraction was selected for analysis[26]. Foraminifera tests were concentrated by flotation using heavy liquid (CCl_4, 1.66 g cm^{-3}) and then picked from a graticuled tray using a stereo microscope.

A total of 30 calcareous and 13 agglutinated benthic species were identified within ER1116. Three species of planktonic species were identified. Robust identification of individual species within a genus was problematic in some instances, especially where unique identifying features are extremely subtle (e.g. *Buccella*, *Elphidium*, and *Islandiella* species). Results are shown from calcareous species that individually accounted for more than 1% of the total assemblage from at least two sampling intervals[27]; these species constitute >98% of calcareous benthic tests.

Foraminifera count, species type, and concentration data are presented in Fig. S4. The data show a dramatic decrease in foraminifera concentrations from ~8000 to 4500 years BP (Fig. S4a). This is followed by a shift at ~4500 years BP from assemblages dominated by calcareous foraminifera to those predominantly composed of agglutinated specimens (Fig. S4b). This shift is also accompanied by a drop in the concentration of planktonic foraminifera. The late-Holocene, from 4000 years BP onwards, is characterised by very low foraminifera concentrations, the dominance of agglutinated species, and the near-absence of planktonic species.

Foraminiferal assemblage zones

Three broad foraminiferal assemblage zones are identified from the data; these are based on the occurrence of key indicator species (Figs. S5, S6, and S4), the dominance of calcareous or agglutinated foraminifera (Fig. S4b), and the concentration of

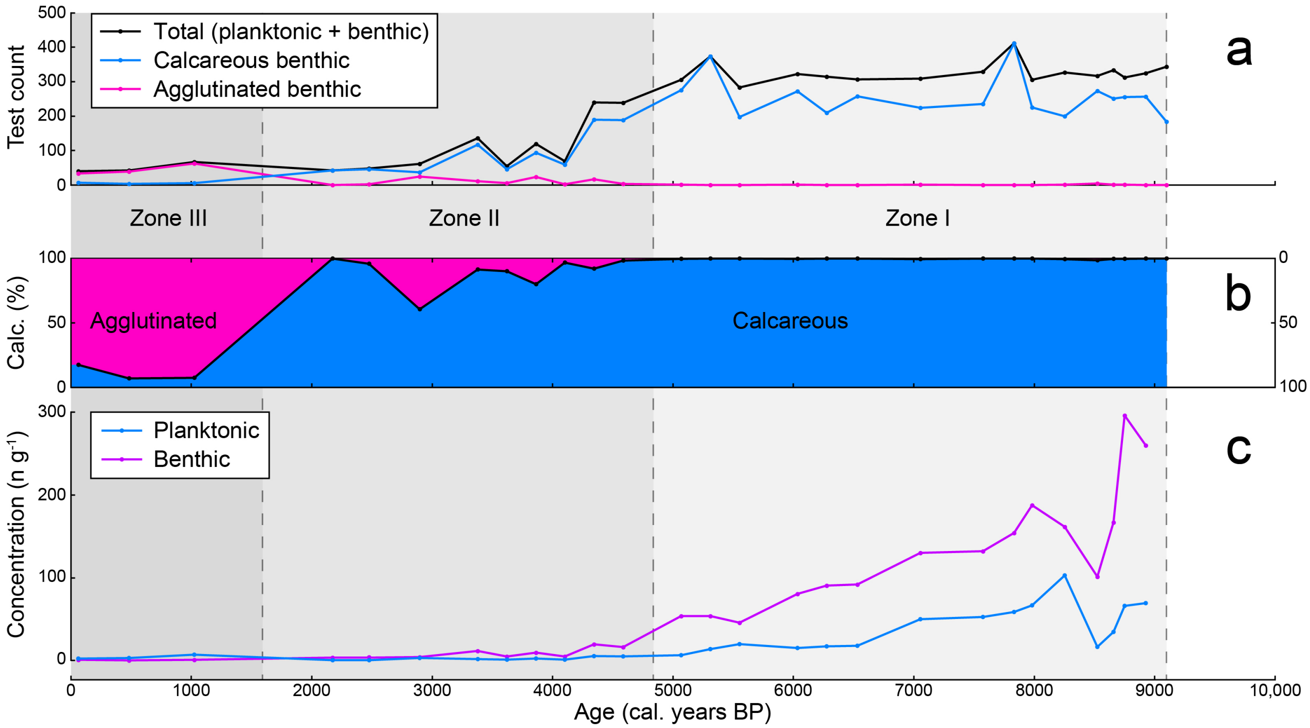

Figure S4. Foraminifera counts, relative percentages, and concentrations. (a) Foraminifera test counts, benthic foraminifera (magenta), and planktonic foraminifera (blue). (b) Relative percentage of calcareous (blue) and agglutinated (magenta) foraminifera. (c) Concentration of foraminifera in core ER1116 (per gram of wet sediment). Concentration data are not available from the base of the core (9120 ±150 cal. years BP). This figure was created using *Microsoft Excel 2013* and *Adobe Illustrator CS6*.

benthic foraminifera (Fig. S4c). The boundaries between zones are arbitrarily drawn at the mid-point between the sampling intervals where a change is observed; the shift in assemblage may occur anywhere within this interval. The palaeo-environmental significance of each foraminifera assemblage zone is discussed below.

Assemblage zone I: 9100 to 4800 years BP

Assemblage zone I was dominated by calcareous species; agglutinated foraminifera were virtually absent from this interval. The total concentration (n g^{-1}) of foraminifera tests was high at the start of this interval and drops progressively (from ∼300 to ∼50 g^{-1}). A substantial dip in foraminifera concentration occurred at ∼8500 years BP (Fig. S4c), but this was not accompanied by an attendant change in the species assemblage; this remained similar to the rest of zone I (Figs. S5 and S6).

The foraminiferal assemblage during this interval was dominated by *Cibicides lobatulus*, *Elphidium* spp., and *Cassidulina neoteretis*. *Islandiella* spp., *Melonis barleeanus*, and *Pullenia bulloides* were notable accessory species (Fig. S5). *C. lobatulus* is widely associated with coarse sediments and high-energy environments[28–33]. Less is known about the environmental preferences of *Elphidium* species. *Elphidium excavatum f. clavata* forms a large proportion of this genus grouping and is generally associated with cooler water masses and glacier proximal conditions[28,30,34,35]. Similarly, *C. reniforme* is considered an Arctic species, and is related to cooler water masses near glacier termini[28–30,35]. *C. neoteretis* has a well-established association with Atlantic water masses and is often viewed as diagnostic of warm, saline oceanographic conditions[29,30,36,37]. This is consistent with the presence of *P. bulloides* which is also linked with warm Atlantic waters[25]. *M. barleeanus* is a detrital feeder that dwells within the sediment matrix (infaunal); it is commonly associated with an abundance of buried organic matter[30,38]. The *Islandiella* genus grouping is composed of *Islandiella helenae* and *Islandiella norcrossi*; both are typical of cool, Arctic shelf conditions and may also be associated with enhanced productivity linked to seasonal sea-ice[25,29,30].

The foraminiferal assemblage data suggest that oceanographic conditions in Køge Bugt from 9100 to 4800 years BP were characterised by elevated hydrodynamic energy levels, the incursion of warm, saline Atlantic water, and possibly also intervals of cooler and fresh Arctic water masses. The foraminifera assemblage data also indicate that Køge Bugt was a glacier proximal environment during this interval and was subject to seasonal sea-ice cover. Finally, the presence of *M. barleeanus* is consistent with a period of enhanced sedimentation in Køge Bugt.

Assemblage zone II: 4800 to 1500 years BP

Assemblage zone II was characterised by a significant reduction in the concentration of benthic foraminifera and an increased, but variable, abundance of agglutinated species (Figs. S4 and S6).

The foraminiferal assemblage is primarily composed of *Elphidium* spp., *Buccella* spp., *M. barleeanus*, and *Islandiella* spp. (Fig. S5). The *Buccella* genus grouping is composed of *Buccella tenerrima* and *Buccella hannai arctica*; these species are associated with sea-ice and enhanced productivity[25]. The accessory species *Nonionella labradorica* and *Trifarina fluens* are also connected to high productivity environments and are often related to the occurrence of seasonal sea-ice or oceanic frontal zones[25,31,37]. A reduction in *C. neoteretis* and *C. lobatulus* abundance through this interval suggests the establishment of cooler, less-energetic hydrographic conditions. Agglutinated species are abundant; this is consistent with a period of cool, Polar oceanic conditions[29,39].

The foraminiferal assemblage data suggest that the oceanography of Køge Bugt during this interval was characterised by cool and fresh Polar water masses, frequent sea-ice cover, periods of relatively rapid sedimentation, and a reduction in hydrodynamic energy levels at the seabed. The data also indicate an oceanographic cooling trend through zone II, with the establishment of cold Arctic conditions by the end of this interval.

Assemblage zone III: 1500 years BP to the present

Assemblage zone III was characterised by the dominance of agglutinated foraminifera (>70%, Fig. S4b) and very low total foraminifera concentrations (<20 g^{-1}, Fig. S4c). This assemblage zone was characterised by the presence of *Ammoglobigerina globigeriniformis*, *Cribrostomoides* spp., *Silicosigmoilina groenlandica*, and a range of other agglutinated species (Fig. S6). There is relatively little detailed information about the environmental preferences of agglutinated Arctic foraminifera. However, agglutinated foraminiferal assemblages are known to occur in areas of the modern Arctic where oceanographic conditions are dominated by cold, low-salinity, Polar water masses[29,39]. *Textularia earlandii* and *Spiroplectammina biformis* are associated with Polar water masses and glacier proximal conditions[29,39]. *Adercotyma glomerata* may be associated with cooled Atlantic water[28,33]. Although very limited, the assemblage of calcareous species is consistent with cold, low-salinity oceanographic conditions (Fig. S5). Foraminifera data generally indicate that the late-Holocene in Køge Bugt was characterised by cold, low-salinity oceanographic conditions.

Sortable silt analysis

Data from laser diffraction analysis are shown in Fig. S8. The raw data (0.3–63 μm) from all intervals exhibited bimodal distributions (Fig. S8 inset). The secondary peak in particle sizes smaller than 10 μm is a common feature in laser diffraction

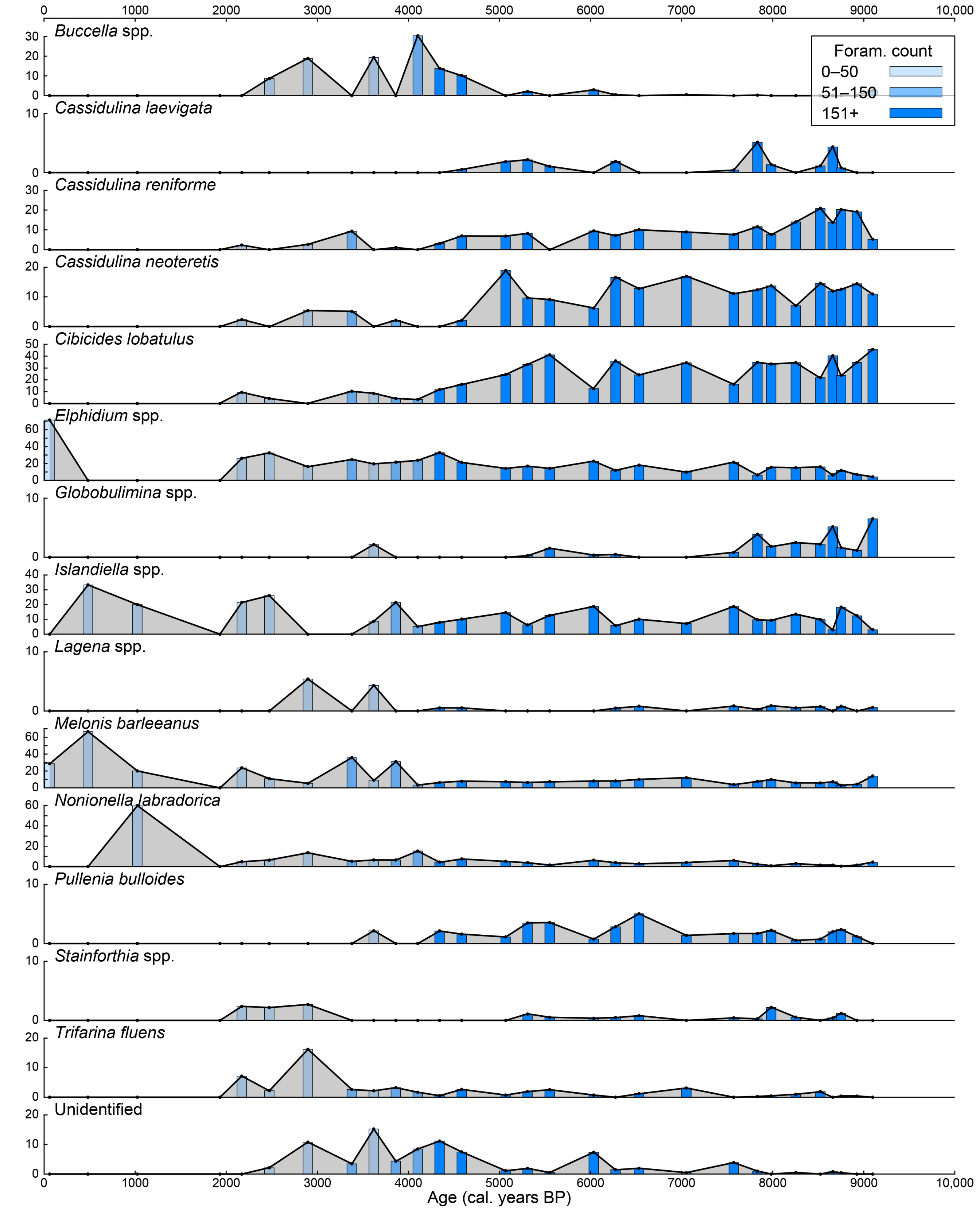

Figure S5. Calcareous benthic foraminifera abundance. Note that the Y-axis scaling varies between plots. Bars are colour coded by the individual sample count of calcareous foraminifera tests. This figure was created using *Microsoft Excel 2013* and *Adobe Illustrator CS6*.

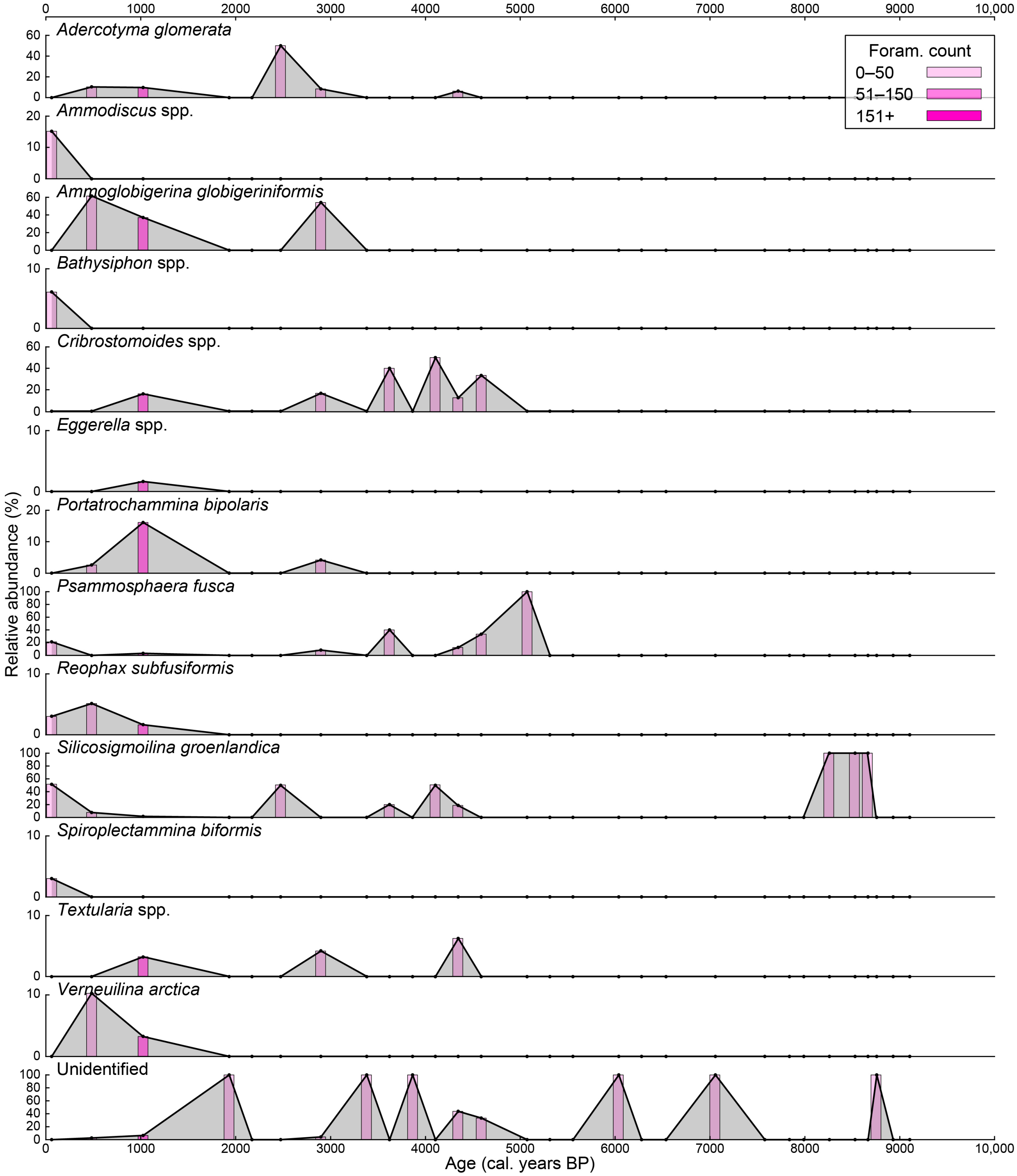

Figure S6. Agglutinated benthic foraminifera abundance. Note that the Y-axis scaling varies between plots. Bars are colour coded by the individual sample count of agglutinated foraminifera tests. This figure was created using *Microsoft Excel 2013* and *Adobe Illustrator CS6*.

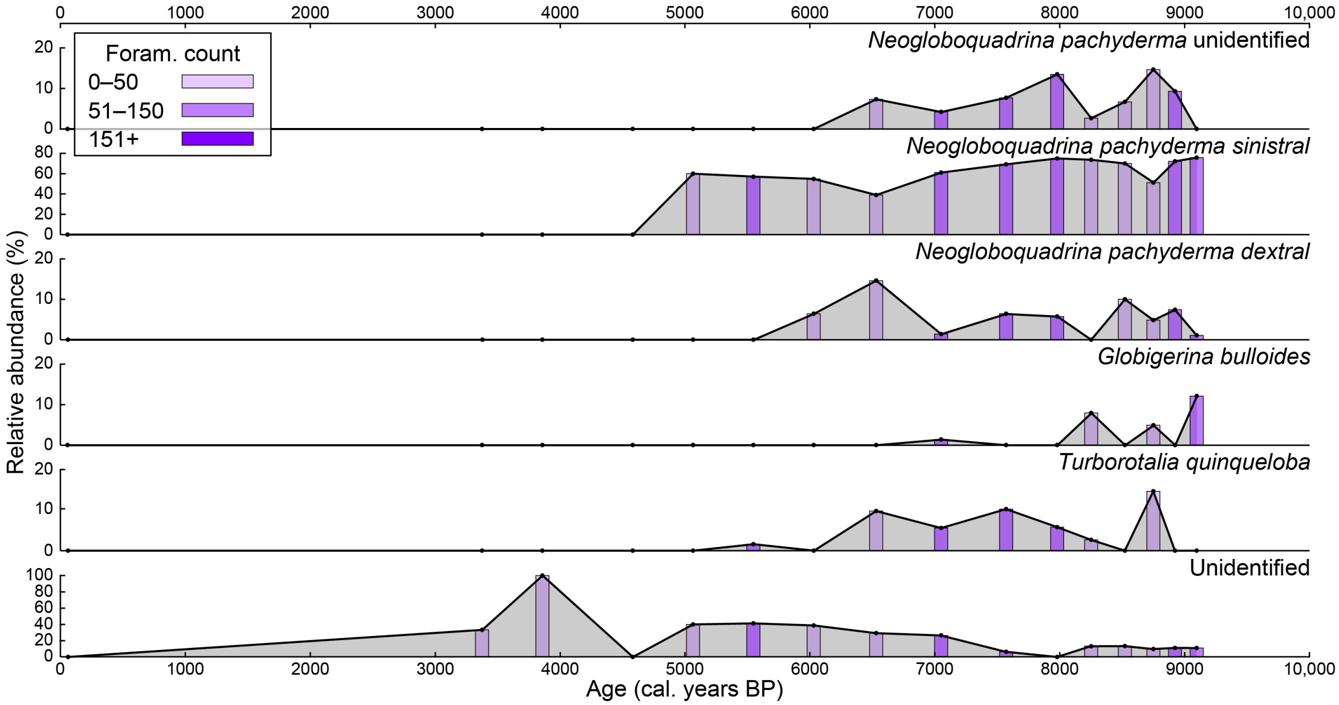

Figure S7. Planktonic foraminifera abundance. Note that the Y-axis scaling varies between plots. Bars are colour coded by the individual sample count of planktonic foraminifera tests. This figure was created using *Microsoft Excel 2013* and *Adobe Illustrator CS6*.

data; this is an artefact caused by the deflocculation of clay mineral aggregates during sample pre-treatment[40,41]. Excluding sediment fractions smaller than 10 μm mitigates this; we focus on the sortable silt fraction (10–63 μm[42]), these data have normal distributions.

Fig. S8 shows both the mean grain size and the standard deviation of the sortable silt fraction. The standard deviation (σ) provides a measure of sorting; small σ values are indicative of a well-sorted sediment. The sortable silt σ can also be used to assess the robustness of laser grain size data as a proxy for palaeocurrent strength. The sortable silt mean grain size and the degree of sorting are weakly correlated (R^2=0.239). However, the longer-term pattern illustrates that periods of high sortable silt mean values (i.e. stronger current) are generally also characterised by a high degree of sorting (Fig. S8). This is consistent with finer material being winnowed out of the sediment and suggests that sortable silt mean data are a viable proxy for benthic palaeocurrent vigour in this setting. The only interval where this relationship fails is from 9000 to 8000 years BP; during this period large sortable silt grain sizes are accompanied by high σ values (Fig. S8). The cause of this remains unclear. We speculate that it may result from the large influx of ice-rafted debris during this interval (Fig. 4c); this may have overwhelmed the current-sorting signal.

References

1. Schjøtt, T. Saqqisikuik: Skjoldungen, 1:500,000. *Saga Maps* **Viking Polar Cruise Series**, 1 (2007).
2. Howat, I. M., Negrete, A. & Smith, B. E. The Greenland Ice Mapping Project (GIMP) land classification and surface elevation datasets. *The Cryosphere* **8**, 1509–1518 (2014).
3. Korsgaard, N. J. *et al.* Digital elevation model and orthophotographs of Greenland based on aerial photographs from 1978–1987. *Scientific Data* **3**, 1–15 (2016).
4. Rosenau, R., Scheinert, M. & Dietrich, R. A processing system to monitor Greenland outlet glacier velocity variations at decadal and seasonal time scales utilizing the Landsat imagery. *Remote Sensing of Environment* **169**, 1–19 (2015).
5. Kelly, M. A. & Lowell, T. V. Fluctuations of local glaciers in Greenland during latest Pleistocene and Holocene time. *Quaternary Science Reviews* **28**, 2088–2106 (2009).
6. Khan, S. A. *et al.* Glacier dynamics at Helheim and Kangerdlugssuaq glaciers, southeast Greenland, since the Little Ice Age. *The Cryosphere* **8**, 1497–1507 (2014).

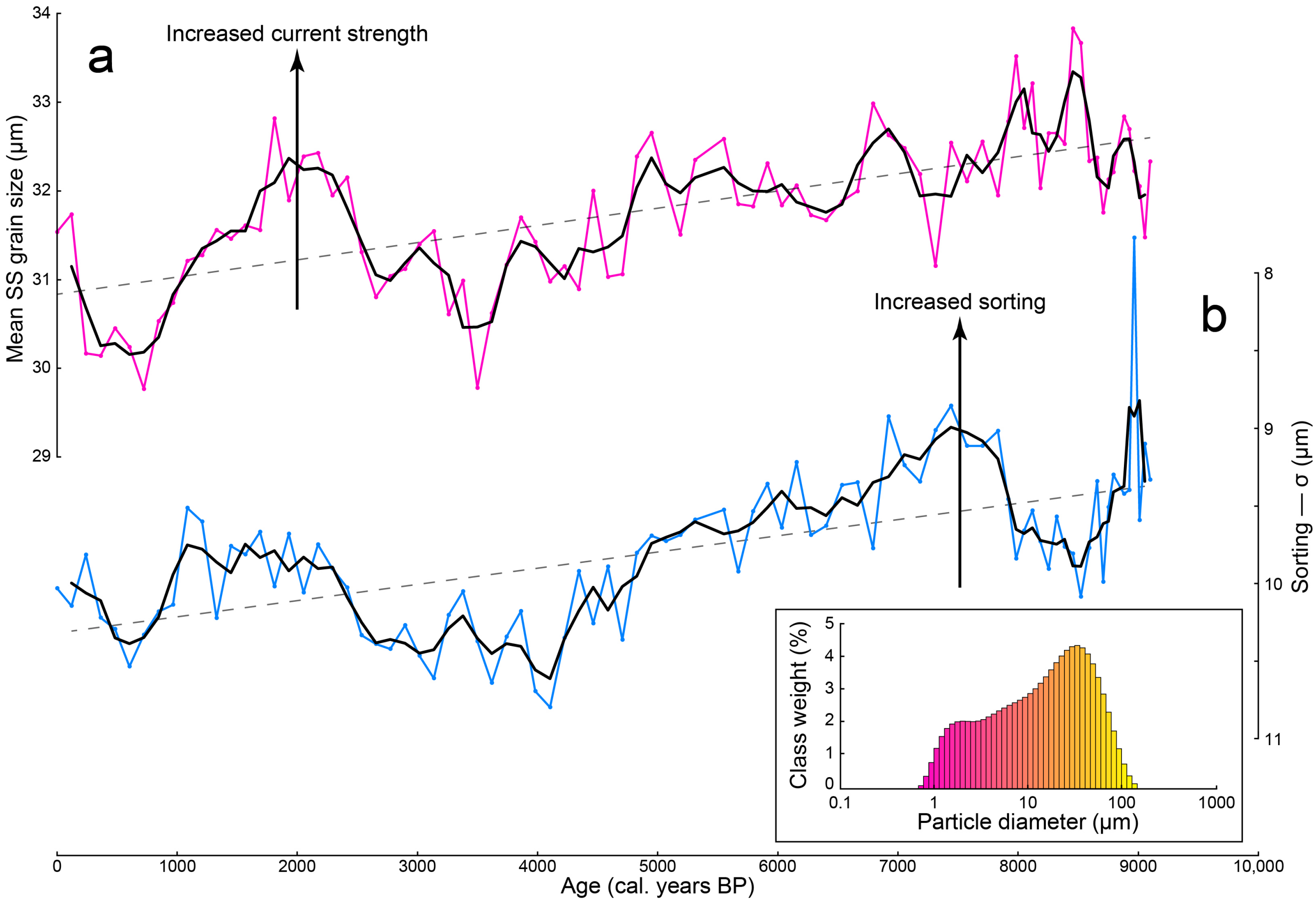

Figure S8. Laser diffraction analysis. (a) The mean sortable silt grain size (μm). (b) The standard deviation (σ) of grain size data from the sortable silt fraction (μm); this provides a measure of the degree of sorting. Black lines show the 3-point running means. Grey dashed lines show the linear trend of each series. The inset shows an example of the bimodal distribution of the raw laser data before filtering (10–63 μm). The sample shown is from 172–173 cm. This figure was created using *Microsoft Excel 2013* and *Adobe Illustrator CS6*.

7. Kjeldsen, K. K. *et al.* Spatial and temporal distribution of mass loss from the Greenland Ice Sheet since AD 1900. *Nature* **528**, 396–400 (2015).

8. Hughes, A. L. C. *et al.* Rapid response of Helheim Glacier, southeast Greenland, to early Holocene climate warming. *Geology* **40**, 427–430 (2012).

9. Dyke, L. M. *et al.* Evidence for the asynchronous retreat of large outlet glaciers in southeast Greenland at the end of the last glaciation. *Quaternary Science Reviews* **99**, 244–259 (2014).

10. Grist, J. P. *et al.* Temperature signature of high latitude Atlantic boundary currents revealed by marine mammal-borne sensor and Argo data. *Geophysical Research Letters* **38**, 1–6 (2011).

11. Andersen, J. M. *et al.* Habitat selection by hooded seals (*Cystophora cristata*) in the Northwest Atlantic Ocean. *ICES Journal of Marine Science: Journal du Conseil* **69**, 1–13 (2012).

12. Sutherland, D. A. *et al.* Atlantic water variability on the SE Greenland continental shelf and its relationship to SST and bathymetry. *Journal of Geophysical Research: Oceans* **118**, 1–9 (2013).

13. Jakobsson, M. *et al.* The International Bathymetric Chart of the Arctic Ocean (IBCAO) Version 3.0. *Geophysical Research Letters* **39**, 1–6 (2012).

14. ICES. The International Council for the Exploration of the Sea Dataset on Ocean Hydrography. Online (2015).

15. Boyer, T. P. *et al.* *World Ocean Database 2013*, vol. 72 (NOAA Atlas NESDIS, 2013). 209 pp.

16. NASA Landsat Program. Landsat 8 OLI scenes LC82330142013246LGN00 and LC82330152013246LGN00, Level 1G, USGS, Sioux Falls (03.09.13) (2013). URL https://earthexplorer.usgs.gov/.

17. Meischner, D. & Rumohr, J. A light-weight, high-momentum gravity corer for subaqueous sediments. *Senckenbergiana maritima* **6**, 105–117 (1974).

18. Syvitski, J. P. M., Stein, A. B., Andrews, J. T. & Milliman, J. D. Icebergs and the Sea Floor of the East Greenland (Kangerlussuaq) Continental Margin. *Arctic, Antarctic, and Alpine Research* **33**, 52–61 (2001).

19. Ó Cofaigh, C. *et al.* Timing and significance of glacially influenced mass-wasting in the submarine channels of the Greenland Basin. *Marine Geology* **207**, 39–54 (2004).

20. Andresen, C. S. *et al.* Rapid response of Helheim Glacier in Greenland to climate variability over the past century. *Nature Geoscience* **5**, 37–41 (2012).

21. Schmidt, S. *et al.* Recent sediment transport and deposition in the Cap-Ferret Canyon, South-East margin of Bay of Biscay. *Deep-Sea Research Part II: Topical Studies in Oceanography* **104**, 134–144 (2014).

22. Stuiver, M. & Reimer, P. J. CALIB 7.10. Online (2016).

23. Reimer, P. *et al.* IntCal13 and Marine13 Radiocarbon Age Calibration Curves 0–50,000 Years cal BP. *Radiocarbon* **55**, 1869–1887 (2013).

24. Jennings, A. E., Hald, M., Smith, M. & Andrews, J. T. Freshwater forcing from the Greenland Ice Sheet during the Younger Dryas: evidence from southeastern Greenland shelf cores. *Quaternary Science Reviews* **25**, 282–298 (2006).

25. Jennings, A., Andrews, J. & Wilson, L. Holocene environmental evolution of the SE Greenland Shelf North and South of the Denmark Strait: Irminger and East Greenland current interactions. *Quaternary Science Reviews* **30**, 980–998 (2011).

26. Seidenkrantz, M.-S. *et al.* Hydrography and climate of the last 4400 years in a SW Greenland fjord: implications for Labrador Sea palaeoceanography. *The Holocene* **17**, 387–401 (2007).

27. Lloyd, J. M., Park, L. A., Kuijpers, A. & Moros, M. Early Holocene palaeoceanography and deglacial chronology of Disko Bugt, West Greenland. *Quaternary Science Reviews* **24**, 1741–1755 (2005).

28. Hald, M. & Korsun, S. Distribution of modern benthic foraminifera from fjords of Svalbard, European Arctic. *The Journal of Foraminiferal Research* **27**, 101–122 (1997).

29. Jennings, A. E. & Helgadottir, G. Foraminiferal assemblages from the fjords and shelf of Eastern Greenland. *The Journal of Foraminiferal Research* **24**, 123–144 (1994).

30. Jennings, A. E., Weiner, N. J., Helgadottir, G. & Andrews, J. T. Modern foraminiferal faunas of the southwestern to northern Iceland shelf: Oceanographic and environmental controls. *The Journal of Foraminiferal Research* **34**, 180–207 (2004).

31. Rytter, F., Knudsen, K. L., Seidenkrantz, M.-S. & Eiríksson, J. Modern distribution of benthic foraminifera on the North Icelandic shelf and slope. *The Journal of Foraminiferal Research* **32**, 217–244 (2002).

32. Sejrup, H.-P., Holtedahl, H., Norvik, O. & Miljeteig, I. Benthonic foraminifera as indicators of the paleoposition of the Subarctic Convergence in the Norwegian-Greenland Sea. *Boreas* **9**, 203–207 (1980).

33. Lloyd, J. M. Modern distribution of benthic foraminifera from Disko Bugt, West Greenland. *The Journal of Foraminiferal Research.* **36**, 315–331 (2006).

34. Korsun, S. & Hald, M. Modern benthic foraminifera off Novaya Zemlya tidewater glaciers, Russian Arctic. *Arctic, Antarctic, and Alpine Research* **30**, 61–77 (1998).

35. Korsun, S. & Hald, M. Seasonal dynamics of benthic foraminifera in a glacially fed fjord of Svalbard, European Arctic. *The Journal of Foraminiferal Research* **30**, 251–271 (2000).

36. Seidenkrantz, M.-S. *Cassidulina teretis* Tappan and *Cassidulina neoteretis* new species (Foraminifera): stratigraphic markers for deep sea and outer shelf areas. *Journal of Micropalaeontology* **14**, 145–157 (1995).

37. Andresen, C. S. *et al.* Mid- to late-Holocene oceanographic variability on the Southeast Greenland shelf. *The Holocene* **23**, 167–178 (2013).

38. Caralp, M. H. Size and morphology of the benthic foraminifer *Melonis barleeanum*; relationships with marine organic matter. *The Journal of Foraminiferal Research* **19**, 235–245 (1989).

39. Hunt, A. S. & Corliss, B. H. Distribution and microhabitats of living (stained) benthic foraminifera from the Canadian Arctic Archipelago. *Marine Micropaleontology* **20**, 321–345 (1993).

40. van Olphen, H. *An Introduction to clay colloid chemistry* (John Wiley and Sons, 1977), second edn. 301 pp.

41. Andresen, C. S. *et al.* A 100-year record of changes in water renewal rate in Sermilik fjord and its influence on calving of Helheim glacier, southeast Greenland. *Continental Shelf Research* **85**, 21–29 (2014).

42. McCave, I. N., Manighetti, B. & Robinson, S. G. Sortable silt and fine sediment size/composition slicing: Parameters for paleocurrent speed and paleoceanography. *Paleoceanography* **10**, 593–610. (1995).
